# Editorial: Targeted cancer therapy through metabolic pathways

**DOI:** 10.3389/fmmed.2026.1842647

**Published:** 2026-05-05

**Authors:** Prasanna Srinivasan Ramalingam, Md Sadique Hussain, Shreyas Ramchandra Gaikwad, Sivakumar Arumugam

**Affiliations:** 1 Protein Engineering Lab, School of Biosciences and Technology, Vellore Institute of Technology, Vellore, Tamil Nadu, India; 2 Uttaranchal Institute of Pharmaceutical Sciences, Uttaranchal University, Dehradun, Uttarakhand, India; 3 School of Pharmaceutical Sciences, Lovely Professional University, Phagwara, Punjab, India; 4 SynVino Inc., Huntsville, AL, United States

**Keywords:** cancer metabolism, cancer therapeutics, drug discovery, precision medicine, targeted therapy

Metabolic reprogramming is widely recognized as a hallmark of cancer, playing a pivotal role in tumor progression, therapeutic resistance, immune evasion, and metastasis (Liu et al., 2025). Aberrant metabolic pathways not only support the rapid proliferation of cancer cells but also create vulnerabilities that can be therapeutically targeted. As cancer cells adapt their metabolic networks to meet increased bioenergetic and biosynthetic demands, these pathways present actionable vulnerabilities for targeted therapeutic intervention (Punnasseril et al., 2025). Recent advances in both experimental and computational approaches, including high-throughput omics technologies, systems biology, and bioinformatics, have greatly expanded our understanding of the metabolic landscape in cancer (Catalano et al., 2025). Integrating mechanistic insights with data-driven discovery holds the key to identifying novel therapeutic targets, predicting treatment response, and designing effective metabolism-based therapies.

From this Research Topic, we aimed to comprehensively explore how targeting dysregulated metabolic pathways can open new avenues for selective and effective cancer therapies by bringing together molecular mechanisms, experimental validation including nano-technological approaches, and bioinformatics-driven discovery. This Research Topic includes eight contributions, five original research articles and three reviews, that illuminate how metabolic reprogramming fuels cancer progression and offers novel therapeutic targets. These works, spanning oncology, genetics, and immunology, underscore vulnerabilities in glycolysis, lipid synthesis, amino acid networks, and immune-metabolic crosstalk, aligning closely with the topic’s focus on precision interventions. This Research Topic of Frontiers in Molecular Medicine is dedicated to all researchers and scientists from all over the world who undertake key research in cancer metabolism and cancer therapeutics. A brief overview of the published articles in our Research Topic are given below.

Yang et al. reviewed how HMG-CoA reductase (HMGCR), central to cholesterol synthesis, drives tumor metabolic shifts by supplying lipids and isoprenoids that activate Hippo, Hedgehog, and MAPK signaling pathways. The study also suggests that HMGCR may influences ferroptosis sensitivity while interacting bi-directionally with TNF-α to promote inflammation and immune escape. Statins show preclinical promise against these effects, though clinical hurdles like dosing toxicity persist, highlighting the need for further investigation of inhibitors targeting the HMGCR-TNF axis (Yang et al.).

Zhan et al. compiled a review how clear cell renal carcinoma’s (ccRCC) reliance on VHL/HIF-driven glycolysis, lipid accumulation, glutamine addiction, and one-carbon metabolism, fostering immunosuppression via TAMs and exhausted T cells. They also advocate HIF-2α antagonists like belzutifan, ferroptosis inducers, and glutamine blockers combined with checkpoint inhibitors, supporting stratified therapy guided by radiomics and lipid metabolic phenotypes (Zhan et al.).

Huang et al. examined LDH isoenzymes, especially LDHA, which is elevated in ovarian, endometrial, and cervical cancers to enable Warburg-effect glycolysis under hypoxia, fueling invasion and acidosis that hampers T cells. Elevated LDH level also correlates with advanced disease stage, metastasis, and chemotherapy resistance, positioning it as a prognostic marker. LDHA blockade inhibits growth preclinically, yet context-specific ferroptosis risks in cervical cases complicate translation (Huang et al.).

He et al. used integrated multi-omics to define bladder cancer subtypes via 144 dysregulated amino acid genes, revealing a poor-prognosis cluster with glutamine/tryptophan hyperdrive, proliferation signals, and inflamed-yet-suppressed immunity. Their 16-gene risk model demonstrate improved performance compared to existing approaches, predicts PI3K/mTOR and immunotherapy responses, and localizes to malignant epithelia, validated by PSPH knockdown curbing invasion (He et al.).

Li et al. revealed CELF1, an RNA-binding protein overexpressed in luminal A (ER-positive) breast cancers, as a driver of aggressive phenotypes through GLUT1-mediated aerobic glycolysis. High CELF1 correlates with poor survival and ESR1 expression, while its knockdown inhibits proliferation, colony formation, migration, and xenograft growth by downregulating glycolytic enzymes HK2 and G6PD alongside cyclin D1. Overexpression in HER2 cells yields opposite effects, while combining CELF1 knockout with GLUT1 inhibitor BAY-876 synergistically suppresses tumors, suggesting this axis as a potential therapeutic target for luminal subtype (Li et al.).

Wang et al. applied WGCNA and machine learning to TCGA-LIHC, pinpointing a glycolysis/lipid module yielding hub genes (e.g., PKM, ACAT2) via LASSO-Cox signatures. High-risk profile predicts recurrence, sorafenib response, and *in vitro* knockdown validates proliferation inhibition. Multi-omics nomogram integrates hubs for prognosis, advocating metabolic panels for hepatocellular therapy stratification (Wang et al.).

Han et al. integrated multi-omics that unveils GABARAP-mediated mitophagy sustaining pyruvate metabolism as osteosarcoma progression drivers. This work also positions mitophagy-pyruvate inhibition, potentially via ATG modulators or PDK blockers as novel metabolic therapies. They also revealed that GABARAP links mitophagy-driven metabolic adaptation with immune evasion, representing a key regulator and potential therapeutic target in osteosarcoma (Han et al.).

Altogether, the articles in this Research Topic reflect the crucial importance of metabolic reprogramming in cancer development, which refers to glycolysis as well as lipids metabolism, use of amino acids and mitophagy, and interactions between immunometabolism. Such studies highlight the role of increasing significance of multi-omics techniques and stratification based on biomarkers to define actionable metabolic targets. Nonetheless, issues including metabolic plasticity, heterogeneity of tumors and its potential toxicity to normal tissues are also a significant obstacle to clinical translation. The next step in the research should be investigating ways of combining metabolic intervention with immunotherapy and precision medicine approaches to increase the effectiveness and sustainability of therapy.
